# Exploring the Lung–Liver Axis in Pulmonary Arterial Hypertension

**DOI:** 10.1002/cph4.70171

**Published:** 2026-05-15

**Authors:** Navneet Singh, Jordan Lawson, Ashok Ragavendran, Somanshu Banerjee, Andy Hon, Alejandro Vega, Jason Hong, Christopher J. Mullin, Mandy Pereira, Allyson Sherman‐Roe, Alexander T. Jorrin, Tiffaney Cayton, Gregory Fishbein, James R. Klinger, William M. Oldham, Rui‐Sheng Wang, Zhiyu Dai, Michael Fallon, Elizabeth O. Harrington, Olin D. Liang, Soban Umar, Corey E. Ventetuolo

**Affiliations:** ^1^ Department of Medicine The Warren Alpert School of Medicine at Brown University Providence Rhode Island USA; ^2^ Center for Advanced Lung Care Brown University Health Providence Rhode Island USA; ^3^ COBRE Center for Computational Biology of Human Disease Brown University Providence Rhode Island USA; ^4^ Department of Anesthesiology University of California Los Angeles California USA; ^5^ Department of Medicine University of California Los Angeles California USA; ^6^ Brown University Health Providence Rhode Island USA; ^7^ Department of Pathology University of California Los Angeles California USA; ^8^ Department of Medicine Brigham and Women's Hospital Boston Massachusetts USA; ^9^ Division of Pulmonary and Critical Care Medicine Washington University School of Medicine St. Louis Missouri USA; ^10^ Department of Medicine Arizona College of Medicine – Phoenix Phoenix Arizona USA; ^11^ Division of Biology and Medicine Brown University Providence Rhode Island USA; ^12^ Department of Health Services, Policy and Practice Brown University Providence Rhode Island USA

**Keywords:** endothelial cell, hepatic fibrosis, hepatic growth factor, model for end stage liver disease, pulmonary arterial hypertension, pulmonary vascular inflammation, Sugen‐hypoxia

## Abstract

The liver's contribution to pulmonary arterial hypertension (PAH) pathogenesis remains unclear. We hypothesized that the liver promotes inflammatory injury to the pulmonary endothelium. PAH patients without liver disease with pulmonary artery endothelial cell (PAEC) biopsies were included. Unsupervised CART analysis of liver serologies identified subclinical dysfunction clusters; machine‐learning models informed differential expression and protein–protein interaction network assembly. PAEC transcriptomes were compared to liver and lung data from monocrotaline and Sugen‐Hypoxia rats. Liver fibrosis was assessed in rat and human PAH livers. Among 25 PAH patients (76% female, median age 61 [30–84] years), CART identified clusters distinguished by Model for End‐Stage Liver Disease Sodium (MELD‐Na) ≥ 12, which was associated with higher Fibrosis‐4 scores and higher pulmonary vascular resistance (ß = 0.5 Wood units per point increase in MELD‐Na, 95% CI 0.2–0.8, *p* = 0.005) after adjustment for right atrial pressure. Subjects with MELD‐Na ≥ 12 had decreased 6‐min walk distance (353 [120–576] m vs. 411 [300–600] m, *p* = 0.03). In comparing the two clusters, a protein–protein interaction network analysis identified IL‐6 as the primary hub of a transcriptional module enriched for leukocyte chemotaxis and myeloid leukocyte migration (all FDR < 0.05) among the High‐MELD‐Na group. Rat livers demonstrated immune activation and a trend toward increased fibrosis (20.8 vs. 16.6% area stained, *p* = 0.09), and human PAH livers without liver disease showed an intermediate fibrotic phenotype between controls and portopulmonary hypertension, though this did not reach statistical significance. Our observations support a lung–liver axis in PAH even in the absence of liver disease, warranting further study.

## Graphical abstract

Among PAH patients without liver disease, subclinical liver dysfunction quantified by MELD‐Na associates with higher pulmonary vascular resistance and inflammatory activation of the pulmonary endothelium, characterized by an IL‐6‐centered transcriptional network enriched for leukocyte chemotaxis. Hepatic inflammation and fibrosis in experimental and human PAH support a lung‐liver axis independent of overt liver disease.

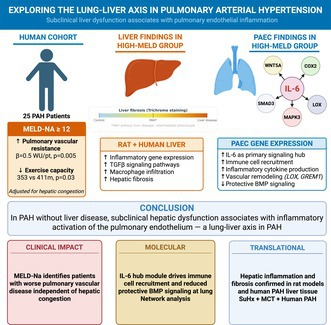

## 1 Introduction

Pulmonary arterial hypertension (PAH) is a progressive and fatal disorder that can be associated with systemic conditions including liver disease. Hepatic congestopathy is a known complication of right ventricular (RV) dysfunction, and portal hypertension has been linked to long‐term prostanoid treatment in PAH (Schoenberg et al. 2022). However, recent work suggests that distinct liver injury phenotypes occur in well‐controlled PAH independent of RV failure and without primary liver disease (Scott et al. 2024). While PAH is increasingly recognized as a multisystem disease, the degree to which pulmonary vascular remodeling is perpetuated by inter‐organ crosstalk in PAH is unknown (Leuchte et al. 2007; Komócsi et al. 2010; Meloche et al. 2017; Batt et al. 2014; Nickel et al. 2017; Potus et al. 2014; Singh et al. 2024).

The liver has several key functions as an immune organ, including the presence of resident macrophages (Kupffer cells), induction of antigen specific tolerance, and local surveillance of gut bacteria (Racanelli and Rehermann 2006). Immunologic and vasoactive factors that first pass through hepatic metabolism (Al‐Naamani and Roberts 2013) and vascular growth factors secreted by the liver (e.g., bone morphogenetic protein [BMP] 9, a central signaling ligand in PAH) may influence distal vascular beds. Hepatic endothelial cells (ECs) secrete cytokines that contribute to left heart failure via coronary EC dysfunction (Salah et al. 2021), suggesting the liver may function as an endocrine organ via EC crosstalk. Despite the liver's role as a regulator of immunity and clinically apparent lung‐liver phenotypes in PAH, it's contribution to pulmonary vascular disease remains unclear.

The current study sought to characterize hepatic involvement in experimental and human PAH without known liver disease. We hypothesized that the liver is associated with systemic inflammation and influences the pulmonary endothelium in PAH. We tested this hypothesis by first using an unsupervised approach to identify subclinical liver dysfunction in PAH patients as determined by laboratory values and Model for End‐Stage Liver Disease with Sodium (MELD) scores, which has been applied in non‐cirrhotic states to predict outcomes in advanced heart failure (Curcio et al. 2025). We then determined the association of cluster membership with pulmonary vascular resistance (PVR) and the differences in differential gene expression and protein–protein interactome networks in human PAH pulmonary artery endothelial cells (PAECs) (Paudel et al. 2025; Singh et al. 2023; Ventetuolo et al. 2020) between the clusters. We compared these results to lung EC gene expression from two experimental pulmonary hypertension (PH) rat models. Finally, we evaluated bulk RNA sequencing of PH rat livers and assessed the degree of hepatic fibrosis both in rats and patients with PAH.

## 2 Methods

### 2.1 Sex as a Biological Variable

Both male and female human participants were included, and sexually dimorphic results are reported. Our study examined male rats only. It is unknown whether the findings are relevant for female rats.

### 2.2 PAH Cohort

Participants with World Symposium on PH Group 1 PAH as diagnosed by a clinical PH provider and PAEC transcriptomic data were included. The clinical diagnosis of PAH was confirmed independently by author N.S. We excluded participants with portopulmonary hypertension (PoPH). All available clinical data (laboratory, imaging, diagnostic codes) were reviewed to confirm the absence of known liver disease defined by elevated transaminases, evidence of synthetic liver dysfunction (elevated international normalized ratio [INR], hypoalbuminemia, thrombocytopenia), or imaging abnormalities including cirrhotic morphology, hepatosplenomegaly or steatosis. The final study cohort consisted of 25 PAH participants (Figure S1).

### 2.3 Human PAEC Biopsies and RNA Sequencing

PAECs were collected at the time of clinically indicated right heart catheterization (RHC) using our previously published cell biopsy method (Paudel et al. 2025; Singh et al. 2023; Ventetuolo et al. 2020). PAECs were cultured from RHC balloon tips, expanded to passage 3 or 4 and submitted for library preparation and bulk RNA sequencing (Azenta, Cambridge, MA). Libraries were sequenced using a 2 × 50 bp paired‐end rapid run on the Illumina HiSeq2500 platform. FastQ files were aligned to the human genome reference sequence GRCh38 using HISAT2 (Kim et al. 2015) and gene IDs were resolved using annotateMyIDs (Dunning et al. 2017).

### 2.4 Cluster Analysis of Clinical Data, and Differential Gene Expression Among Clusters

The medical record was then retrospectively reviewed, and data including invasive hemodynamics, all available liver imaging and laboratory data (liver transaminases, albumin, platelets, total bilirubin, sodium, creatinine and INR) were collected. The MELD sodium (MELD‐Na) 3.0 (Kim et al. 2021) and MELD Xi (Wernly et al. 2017) were calculated and included as independent clinical variables. Clinical data and laboratory values were collected as close as possible to the date of PAEC biopsy and within 6 months of biopsy/RHC catheterization date. An unsupervised classification and regression tree (CART) (Chen et al. 2011) was used to identify distinguishable clusters within the data. Linear regression was subsequently used to model the relationship between these CART‐derived clusters and PVR at the time of PAEC cell biopsy, adjusting for right atrial pressure. Linear regression was performed to assess if any of the individual components of MELD‐Na contributed to the relationship between PVR and the composite score. Sensitivity analyses were performed to evaluate the influence of connective tissue disease patients and those taking warfarin on the results. The Fibrosis‐4 (FIB‐4) (Sterling et al. 2006; Shah et al. 2009) index was calculated for each patient to provide orthogonal validation of identified clusters.

To understand the possible biological differences between clusters, differentially expressed genes of these subjects' PAECs were generated using linear discriminant analysis (LDA) with principal component analysis (PCA), LDA with weighted gene co‐expression analysis (WGCNA), and a random forest model (Zhao et al. 2024; Greenacre et al. 2022; van Dam et al. 2018). All three models were assessed for accuracy to predict group assignment. Gene lists generated by all three models were analyzed using Ingenuity Pathway Analysis (Qiagen); significance for pathway enrichment was calculated using a right‐tailed Fisher's Exact Test and gene lists and directional effects predicted using the Ingenuity Knowledge Base.

### 2.5 Protein–Protein Interaction Network Analysis

To identify functional relationships between differentially expressed genes and uncover the signaling pathways and molecular mechanisms underlying the transcriptomic differences between High‐ and Low‐MELD clusters, genes with nominal *p* < 0.05 (*n* = 2458) were carried forward for protein–protein interaction (PPI) network analysis. PPI networks model physical and functional interactions between gene products, enabling identification of coordinated biological programs that would not be apparent from gene lists alone (Maron et al. 2021; Wang and Loscalzo 2021). A network was constructed with STRINGdb (v2.16.4, combined confidence threshold ≥ 400) and analyzed using igraph (v2.2.2) to derive node‐level topology metrics including degree, betweenness centrality, and interaction strength. Network community structure was identified using the Louvain modularity optimization algorithm to detect functional modules—groups of genes with dense interactions that reflect shared biological function or pathway membership (Blondel et al. 2008). Betweenness centrality was calculated for each node to identify hub genes: those that serve as critical bridges connecting otherwise disparate regions of the network, reflecting their disproportionate role in coordinating signaling between functional modules (Abedi and Gheisari 2015). Gene Ontology (GO) Biological Process enrichment analysis was performed on the primary hub module using clusterProfiler (v4.12.6) with Benjamini‐Hochberg false discovery rate (FDR) correction; terms with FDR < 0.05 were considered significant. Full methodological details are provided in the Supplement.

### 2.6 Bulk RNA Sequencing of Sugen‐Hypoxia and Monocrotaline Livers

To validate findings of hepatic dysfunction in human PAH, we next turned to two leading animal models of PH. The Sugen‐hypoxia (SuHx) and monocrotaline (MCT) rat models were performed as described extensively in prior publications by our groups (Klinger et al. 2020; Banerjee et al. 2022). The right lobe of the liver was removed and fixed in formalin, paraffin embedded, sectioned at 5 μm and stained with Masson's trichrome per standard protocol. All images were acquired using a confocal microscope (Nikon) with a minimum of three images acquired from each slide. Fibrosis was quantified using the Otsu algorithm in Fiji (Image J; NIH). Total RNA was isolated from flash frozen livers from SuHx, MCT, and control rats (four each, all male) using Trizol (Invitrogen). RNA samples were submitted for bulk RNA sequencing to the sequencing core of the University of California, Los Angeles (UCLA); differentially expressed gene lists were generated using DESeq in R (Love et al. 2014) and then analyzed using Ingenuity Pathway Analysis (Qiagen) where the significance of enrichment for pathways was calculated using a right‐tailed Fisher's Exact Test.

### 2.7 Single‐Cell RNA Sequencing of Sugen‐Hypoxia and Monocrotaline Lungs

To further validate our human PAEC findings, we utilized a publicly available data set of single‐cell RNA sequencing of SuHX, MCT and control rat lungs (Hong et al. 2021). Raw expression data were normalized, filtered, and clustered using the Seurat R package (Butler et al. 2018). Cell types were identified using the labels assigned by the study authors. Differential gene expression in endothelial and immune cell populations was determined using the Wilcoxon rank‐sum test. Gene expression was subsequently analyzed using Ingenuity Pathway Analysis (Qiagen).

### 2.8 Human Liver Biopsies and Masson's Trichrome Staining

To explore if findings from rat livers were consistent with human disease, we completed complimentary histologic analysis of human tissue from PAH and control patients. Human liver tissue was obtained via warm autopsy at UCLA. Formalin‐fixed paraffin‐embedded 5 μm thick human liver sections (connective tissue disease‐associated PAH [CTD‐APAH] *n* = 3, idiopathic PAH *n* = 1, PoPH *n* = 5, control *n* = 3) were used for Masson's Trichrome staining following standard protocols and quantified using the same methodology as rat livers (Otsu algorithm in Fiji). All images were acquired using a confocal microscope (Nikon). A total of five to ten images were acquired from each slide.

### 2.9 Statistics

Statistical analysis was performed in R (R Core Team 2024), RStudio (RStudio Team 2025), GraphPad Prism 10.6.1 (GraphPad Software), and Ingenuity Pathway Analysis (Qiagen). Linear regression was performed to evaluate the relationship between MELD and its individual components and PVR as described above. Student's *t*‐test and Kruskal–Wallis test with post hoc pairwise comparisons were used to compare the percent area stained of Masson's trichrome in rat and human livers, respectively. Significance for bulk gene expression pathway enrichment was calculated using a right‐tailed Fisher's Exact Test. For single cell data, differential gene expression was determined using the Wilcoxon rank‐sum test.

### 2.10 Study Approval

Collection of PAECs and clinical data was approved by the Institutional Review Board at Brown University Health (IRB# 001218 and 2221835). For human liver tissue, in accordance with 45 CFR 46, acquisition of the tissue did not constitute human subjects research and used deidentified human tissues provided by UCLA Pathology departmental honest broker, the UCLA Translational Pathology Core Lab (IRB# 11–002504). Animal experiments were approved by the Institutional Animal Care and Use Committee at Brown University Health (IACUC# 2207800).

## 3 Results

### 3.1 PAH Patients With High‐MELD Scores Have Worse Clinical Outcomes

Characteristics of the PAH participants with PAECs available are included in Table 1. CART analysis identified that MELD‐Na was predictive of PVR. A MELD‐Na ≥ 12 was associated with a significantly higher PVR after adjustment for right atrial pressure (i.e., degree of hepatic congestion) (ß = 0.5 Wood units per one point increase in MELD‐Na, 95% CI 0.2–0.8, *p* = 0.005). Nine participants (36%) had a MELD‐Na ≥ 12 (High‐MELD group) and 16 (64%) had a MELD‐Na < 12 (Low‐MELD group). Both groups were similar in age, distribution of race and ethnicity, and renal function. Compared to the High‐MELD group, the Low‐MELD group had a higher proportion of female patients (94% vs. 44%), fewer connective‐tissue disease‐associated PAH (CTD‐APAH) patients (31% vs. 44%) and were less likely to be on anticoagulation (0% vs. 33%). Linear regression of the individual variables of MELD‐Na revealed that no single variable in the composite score (e.g., creatinine, INR) was responsible for the relationship with PVR. There was no difference in body mass index (BMI), and there was no evidence of hepatic steatosis on available liver imaging (*n* = 15) indirectly suggesting that hepatic steatosis was not present in this cohort. The six‐minute walk distance (6MWD) was higher in the Low‐MELD group compared to the High‐MELD group (411 m vs. 353 m, *p* = 0.03). Because connective tissue diseases are associated with systemic inflammation (Son and Moon, 2025; Didier et al. 2018) and warfarin can increase the INR (a component of MELD‐Na), we then performed sensitivity analyses excluding patients with connective tissue disease (*n* = 9) and taking warfarin (*n* = 2) and found the relationship between MELD‐Na and PVR was unchanged. The FIB‐4 index was significantly higher in the High‐MELD group (2.3 vs. 1.6, *p* = 0.02), MELD scores correlated with FIB‐4 indices (Spearman ρ = 0.4, *p* = 0.045, Figure S2A) and there was a trend toward higher PVR in individuals with a FIB‐4 ≥ 2.67 (an established clinical threshold for liver stiffness, Shah et al. 2009, Figure S2B).

**TABLE 1:** Participant characteristics.

	Low‐MELD‐Na	High‐MELD‐Na	All PAH participants
	(*n* = 16)	(*n* = 9)	(*n* = 25)
Age, years	52 (30–75)	68 (43–84)	61 (30–84)
Female sex, *n* (%)	15 (94)	4 (44)	19 (76)
Race, *n* (%)			
Black	1 (6)	1 (11)	2 (8)
White	14 (88)	7 (78)	21 (84)
Asian	1 (6)	0	1 (4)
Other	0	1 (11)	1 (4)
Hispanic Ethnicity, *n* (%)	2 (13)	1 (11)	3 (12)
Body mass index (kg/m^2^)	31 (16–40)	35 (23–45)	21 (16–45)
*PAH etiology, n (%)*			
Idiopathic PAH	5 (31)	3 (33)	8 (32)
Hereditary PAH	3 (19)	0	3 (12)
Connective tissue disease‐APAH	5 (31)	4 (44)	9 (36)
HIV‐APAH	1 (6)	0	1 (4)
Congenital heart disease‐APAH	2 (13)	0	2 (8)
PVOD	0	2 (22)	2 (8)
Functional Class III or IV, *n* (%)	3 (30)	2 (40)	5 (20)
	*n* = 10	*n* = 5	*n* = 15
6MWD (m)	411 (300–600)	353 (120–576)	380 (120–600)
*Hemodynamics*			
RAP, mmHg	9 (3–19)	10 (4–15)	10 (3–19)
mPAP, mmHg	41 (22–69)	44 (26–62)	44 (22–69)
PAWP, mmHg	13 (6–15)	11 (6–17)	11 (6–17)
PVR, Wood units	4.6 (1.2–16.8)	6.7 (2.2–19.6)	5.3 (1.2–19.6)
CO, L/min	5.4 (3.3–9.9)	5.3 (1.9–11)	5.3 (1.9–11)
MELD‐Na	9 (7–11)	15 (12–28)	10 (7–28)
FIB‐4	1.6 (0.4–3.35)	2.3 (1.2–6.3)	1.7 (0.4–6.3)
Creatinine (mg/dL)	0.78 (0.52–1.26)	0.96 (0.60–1.65)	0.9 (0.52–1.65)
Anticoagulation, *n* (%)	0 (0)	3 (33)	3 (33)

*Note:* Data are represented as *n* (%) or median (range).

Abbreviations: 6MWD = six‐minute walk distance, CO = cardiac output, FIB‐4 = Fibrosis‐4 index, HIV = human immunodeficiency virus, MELD‐Na = model for end‐stage liver disease with sodium, mPAP = mean pulmonary artery pressure, PAH = pulmonary arterial hypertension, PAWP = pulmonary artery wedge pressure, PVOD = pulmonary veno‐occlusive disease, PVR = pulmonary vascular resistance, RAP = right atrial pressure.

### 3.2 PAECs From High‐MELD Patients Differentially Upregulate Genes Related to Inflammation

**FIGURE 1:**
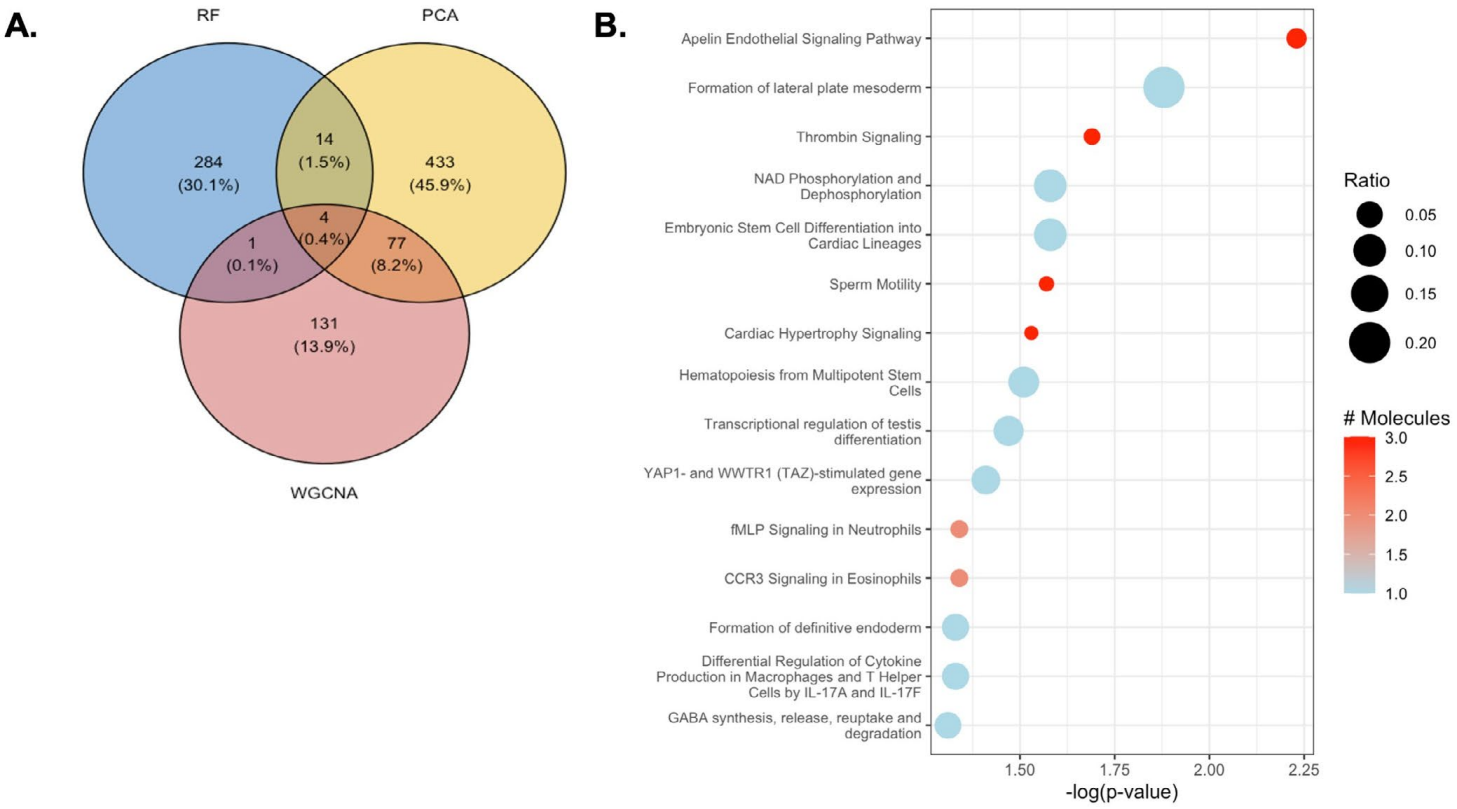
Differences in gene expression from bulk RNA sequencing of pulmonary artery endothelial cell biopsies from PAH participants in the High‐ versus Low‐MELD clusters were examined using three modeling approaches. (A) The number and proportion of genes generated by three independent models (linear discriminant analysis (LDA) with principal component analysis (PCA), LDA with weighted gene co‐expression analysis (WGCNA), and a random forest (RF) model) and their intersections was reviewed and the intersection of LDA with PCA and WGCNA was used for all subsequent analysis. (B) Pathways analysis demonstrates enrichment for pathways related to apelin signaling, YAP/TAZ, as well as immunity and inflammation.

We then used three machine learning models (LDA with PCA, LDA with WGCNA, and a random forest model) to determine gene expression that predicted group assignment (High‐ vs. Low‐MELD); this yielded an accuracy of 0.80, 0.72, and 0.60, respectively. To increase rigor, the union of LDA with PCA and LDA with WGCNA was chosen to build a differentially expressed gene list comparing High‐ vs. Low‐MELD clusters (Figure 1A). Pathways analysis demonstrated enrichment for pathways related to apelin signaling, YAP/TAZ, as well as immunity and inflammation in the High‐MELD group (Figure 1B).

### 3.3 Network Analysis Supports Inflammatory Activation of the Pulmonary Endothelium in High‐MELD Subjects

**FIGURE 2:**
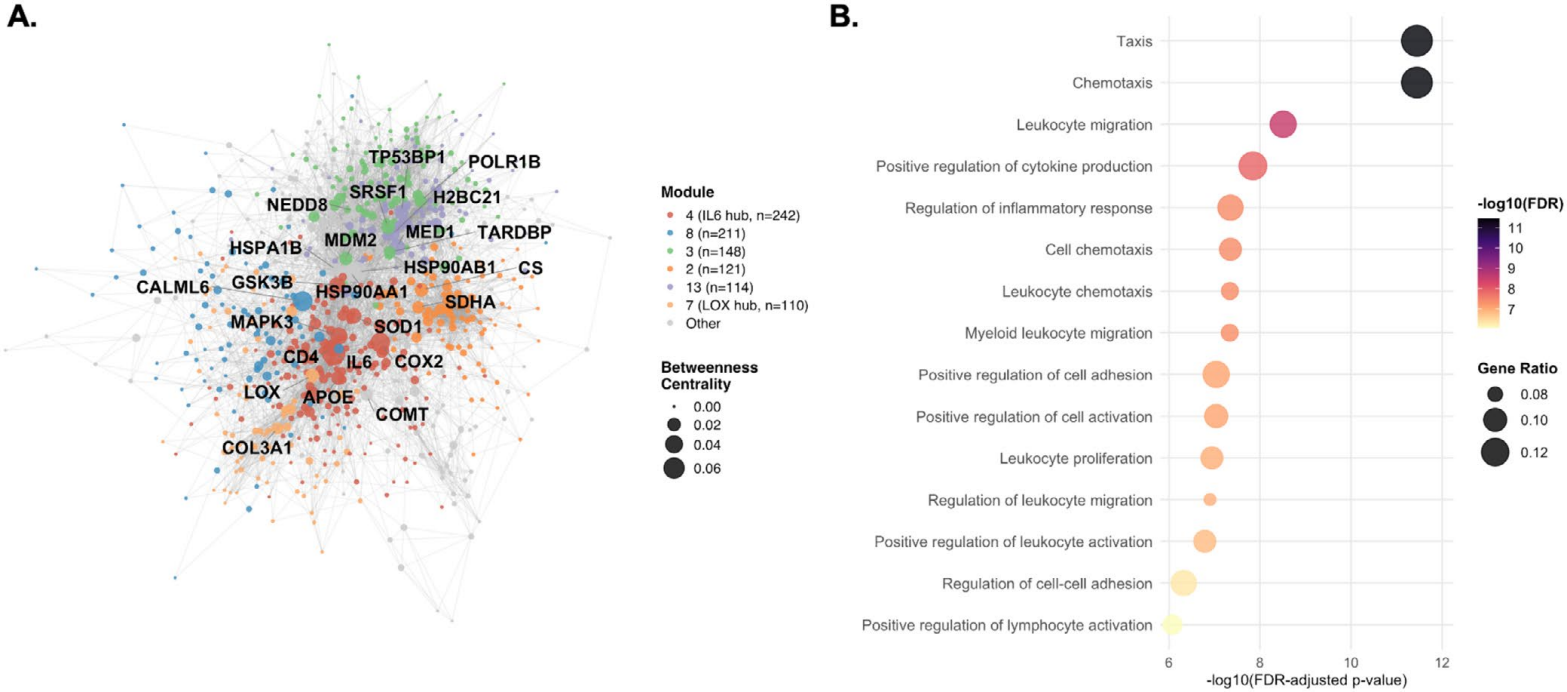
Protein–protein interaction (PPI) network analysis of differentially expressed genes in pulmonary artery endothelial cell biopsies from High‐ versus Low‐MELD‐Na PAH patients identifies IL‐6 as a primary hub of inflammatory activation. (A) PPI network of 480 genes (degree ≥ 5) constructed from 2458 genes with nominal *p* < 0.05 by DESeq2 using STRING (combined confidence score ≥ 400). Node size reflects betweenness centrality; node color reflects Louvain community membership (16 modules, modularity *Q* = 0.554). *IL‐6* emerged as the primary network hub (betweenness centrality = 0.072, degree = 132, log_2_FC = +1.30), co‐localizing in Module 4 (*n* = 242 genes, 81% upregulated in High‐MELD) with genes involved in inflammatory signaling (*COX2, MAPK3*), vascular remodeling (*LOX*), non‐canonical Wnt activation (*WNT5A*), and TGFβ/Activin signaling (*SMAD3, INHBA*). (B) GO Biological Process enrichment of Module 4 demonstrates significant enrichment for leukocyte chemotaxis, myeloid leukocyte migration, positive regulation of cytokine production, and regulation of inflammatory response (all FDR < 0.05, Benjamini–Hochberg correction), consistent with an IL‐6‐centered transcriptional program associated with recruitment and activation of myeloid leukocytes to the pulmonary endothelium.

To move beyond individual gene expression differences and to identify the functional modules and signaling relationships coordinating transcriptomic changes in High‐MELD PAECs, a PPI network was constructed from the 2458 differentially expressed genes. This analysis identified *IL‐6* as the primary network hub (betweenness centrality = 0.072, degree = 132) upregulated in High‐MELD patients (log_2_FC = +1.30) and co‐localizing in a module with genes involved in inflammatory signaling (*COX2, MAPK3*), oxidative stress (*SOD1, NOX1, GPX3*), vascular remodeling (*MMP13, TIMP1*), endothelial‐to‐mesenchymal transition (*THY1*), and non‐canonical Wnt activation (*WNT5A*) (Figure 2A, Tables S1 and S2). GO biological process enrichment of this module demonstrated leukocyte chemotaxis, myeloid leukocyte migration, and positive regulation of cytokine production were significantly enriched (all FDR < 0.05), consistent with IL‐6‐induced recruitment of myeloid leukocytes to the pulmonary endothelium (Figure 2B). A second co‐expression module centered on *LOX* (log_2_FC = +1.58) contained genes related to extracellular matrix remodeling and fibrosis, including *COL3A1* (log_2_FC = +4.20), *LUM*, and *TIMP3*, and co‐localized with the BMP antagonist *GREM1* (log_2_FC = +3.03) and the Activin A subunit *INHBA* (log_2_FC = +1.13), encoding ligands in the TGFß/Activin superfamily. *SMAD3* (betweenness centrality = 0.014, degree = 46, log_2_FC = −0.56), the primary intracellular transducer of TGFß/Activin signaling, co‐localized in the IL‐6 module and was directly connected to *IL‐6*, *WNT5A* and *MAPK3* within the network. *SKIL*, encoding the SMAD transcriptional repressor SnoN, was downregulated (log_2_FC = −0.73) and connected to *SMAD3* with high confidence (combined interaction score = 998/1000).

### 3.4 Liver Histology and Gene Expression From Two PH Rat Models Recapitulates Increased Inflammatory Signaling

**FIGURE 3:**
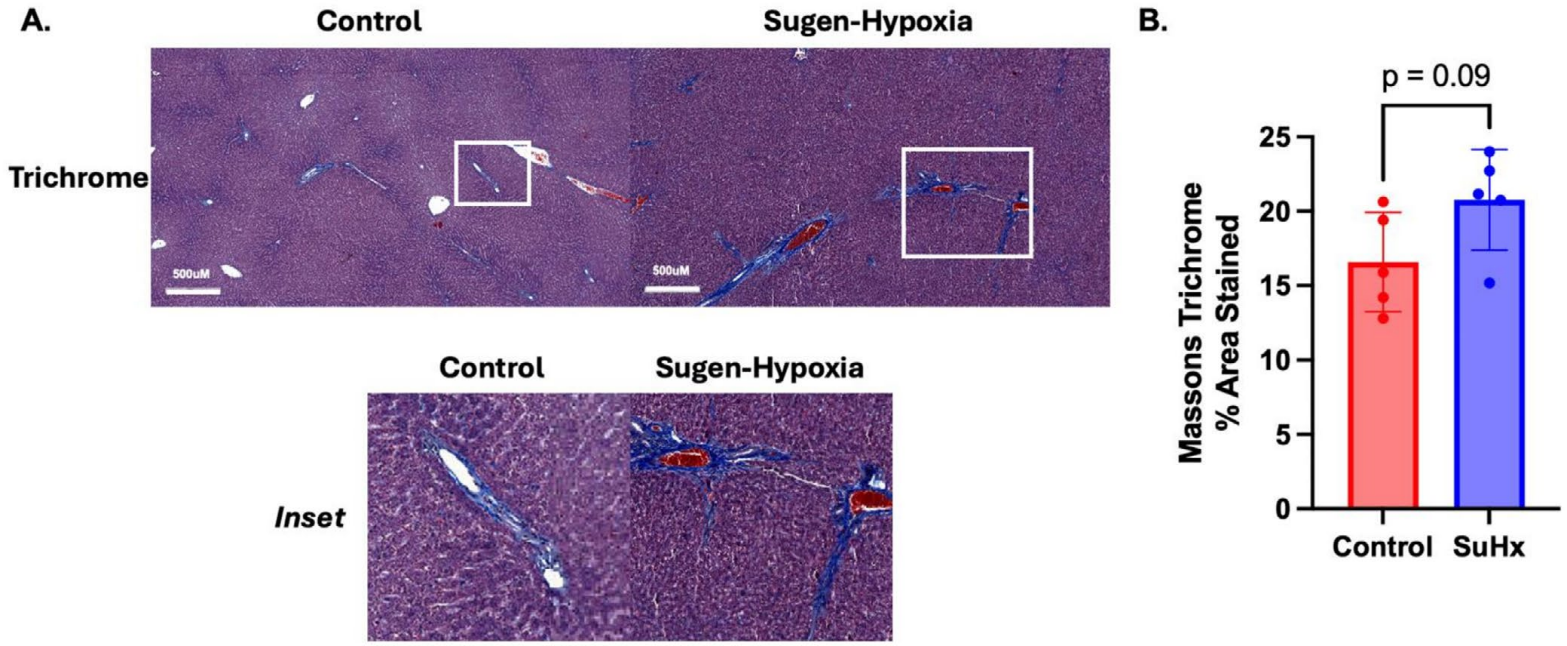
Increased hepatic fibrosis in SuHx rats compared to controls. Control and SuHx rat livers were stained with Masson's Trichrome which resulted in increased perivascular and parenchymal fibrosis (A), 20.8 versus 16.6% area stained, *p* = 0.09 (B). *n* = 5 rats per group. SuHx = Sugen‐hypoxia. All images taken at 4×. Scale bar = 500 μm. Collagen‐positive (blue) area was quantified using the Otsu algorithm in Fiji by a reviewer blinded to group assignment.

**FIGURE 4:**
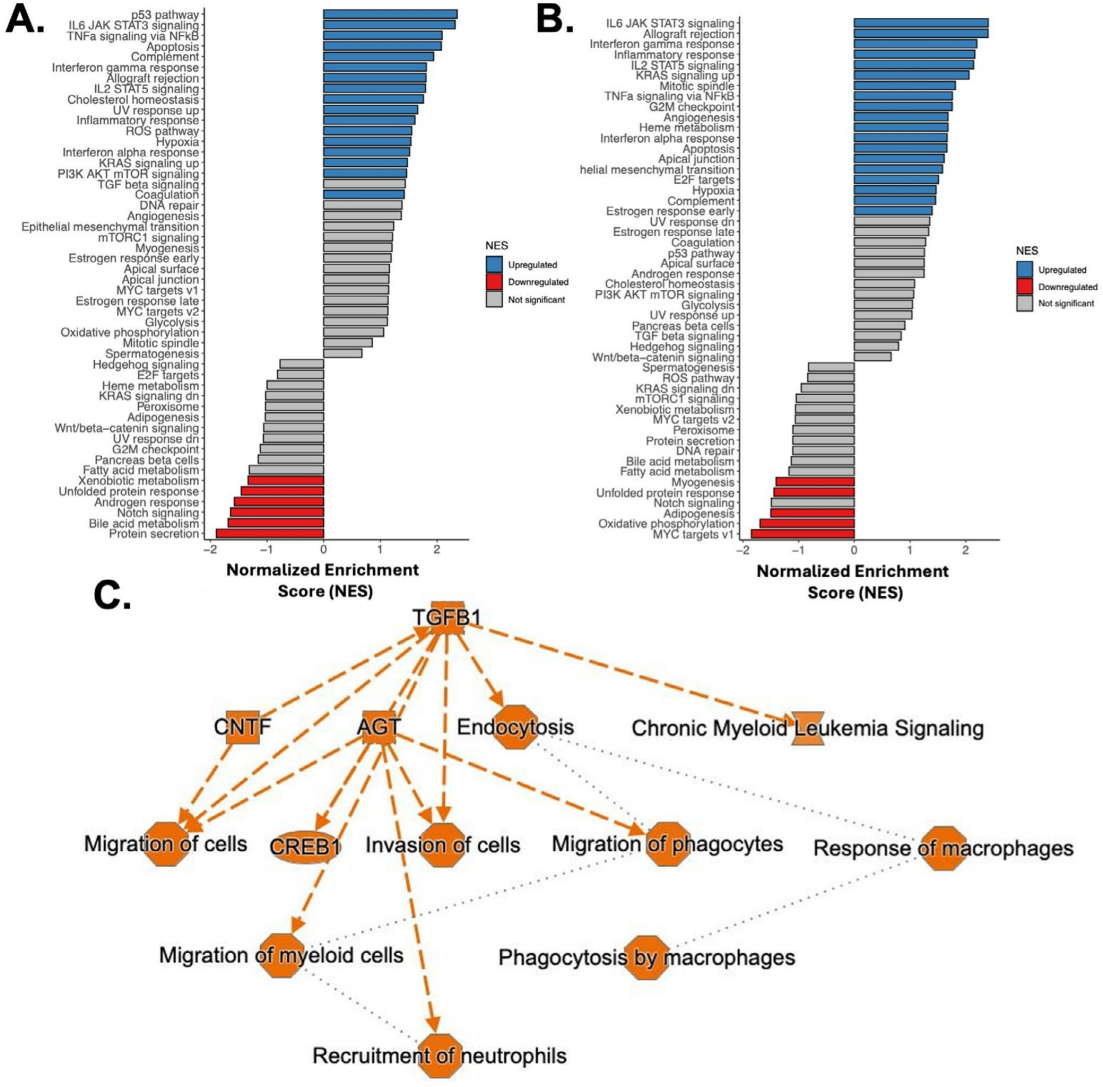
Bulk RNA sequencing of MCT (A) and SuHx (B) male rat livers compared to controls demonstrates increased gene expression in pathways related to inflammation and TGB beta signaling and decreased expression in pathways related to cellular metabolism. (C) A regulatory network built from differentially expressed genes in both MCT and SuHx rat livers demonstrates activation of pathways related to activation and recruitment of leukocytes.

Directed by our network analysis, we next examined whether a similar pro‐inflammatory signature existed in the livers of two small animal models of PH. Trichrome staining revealed a trend toward increased perivascular and parenchymal fibrosis in SuHx rat livers compared to controls (20.8 vs. 16.6% area stained, *p* = 0.09) (Figure 3A,B). Bulk RNA sequencing of SuHx and MCT rat livers demonstrated similar findings to the High MELD‐cluster gene expression data from human PAH PAECs. Specifically, we observed increased expression in pathways related to inflammation and TGFβ signaling and decreased expression in pathways related to cellular metabolism. Although the MCT model is known to be characterized by hepatic inflammation and fibrosis (Copple et al. 2003), these themes were recapitulated in the SuHx model (Figure 4A,B). A regulatory network demonstrated activation of pathways related to activation and recruitment of leukocytes in these experimental PH livers (Figure 4C).

**FIGURE 5:**
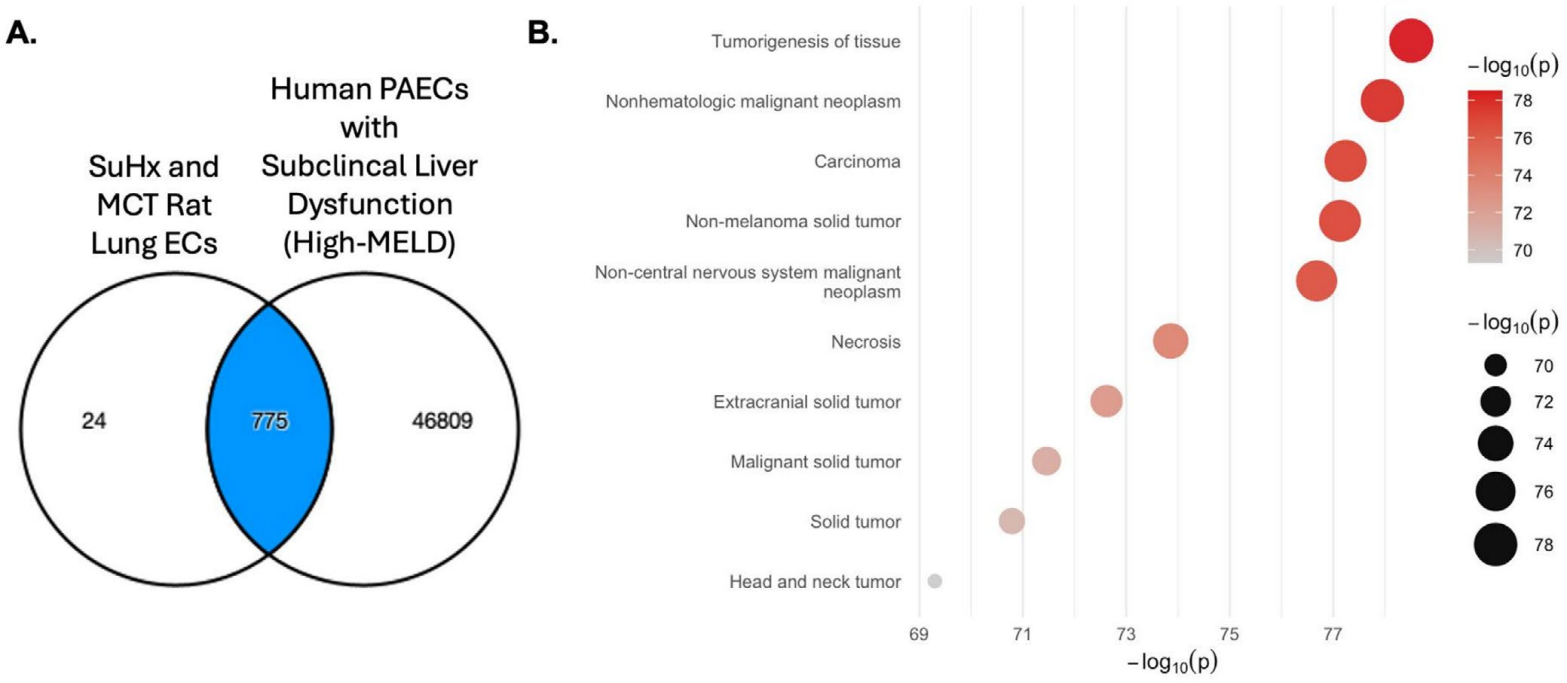
Differential gene expression in SuHx and MCT rat lung endothelial cells from a publicly available single‐cell RNA sequencing dataset was compared to pulmonary artery endothelial cell (PAEC) transcriptomes from High‐MELD participants. (A) Venn diagram demonstrating 775 genes overlapping between human High‐MELD PAECs and rat lung endothelial cells across both models. (B) GO pathway enrichment of the 775 overlapping genes demonstrated significant enrichment for pathways related to cell survival, proliferation, and cancer biology, including *EGR1, ECM1*, and *MIF*. SuHx = Sugen‐Hypoxia. MCT, monocrotaline; MELD, model for end state liver disease.

To validate our observations in humans with PAH with the known biology of these two animal models, differential gene expression in human PAH PAECs from the High‐MELD cluster and single cell sequencing data from SuHx and MCT rat lung ECs were then compared. There were 775 genes overlapping in both data sets (Figure 5A). Three genes were significantly expressed in both humans and animal regulatory networks: early growth response 1 (*EGR1*), extracellular matrix protein 1 (*ECM1*), and macrophage inhibitory factor (*MIF*). The union of these data sets represented genes with high relevance to cancer biology (Figure 5B).

### 3.5 Human PAH Livers Demonstrate Increased Fibrosis

**FIGURE 6:**
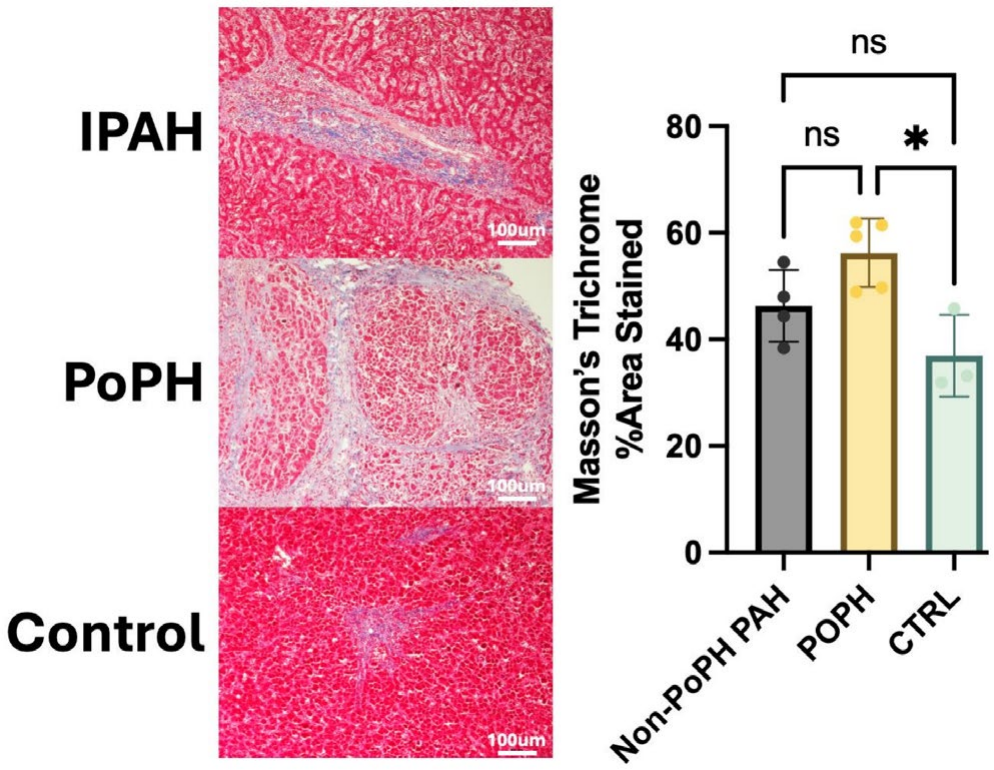
Masson's trichrome staining of liver tissue from humans with and without pulmonary arterial hypertension (PAH) demonstrates that livers from PAH patients without liver disease (non‐PoPH PAH *n* = 4 of which CTD‐APAH *n* = 3, idiopathic PAH *n* = 1) demonstrated quantitatively intermediate fibrosis between controls (CTRL) and portopulmonary hypertension (PoPH). All images taken at 10×. Scale bar = 100 μm. *p = 0.03, ns = not significant.

Finally, we examined human liver tissue from 12 individuals to determine if the hepatic fibrosis seen in SuHx livers was also present in PAH patients with and without liver disease as compared to controls. Perivascular and parenchymal fibrosis by trichrome staining differed across the three groups (H = 7.395, *p* = 0.01). Five PAH patients with liver disease (PoPH) had significantly increased fibrosis as compared to three control patients (56.3 vs. 37.0%, *p* = 0.03). Fibrosis levels in four PAH patients (*n* = 3 CTD‐APAH, *n* = 1 idiopathic PAH) without liver disease had more fibrosis quantitatively (46.3%) and were intermediate between controls and PoPH patients, but these differences were not statistically significant (Figure 6).

## 4 Discussion

We demonstrate evidence of a lung‐liver axis in PAH independent of primary liver disease in both human PAH tissues and two small animal models. Specifically, in an unsupervised cluster analysis, the MELD‐Na score was predictive of PVR among PAH patients with no clinical liver disease and independent of elevated right atrial pressure. In PAECs from patients in the High‐MELD cluster, i.e., those with subclinical liver dysfunction, there was upregulation of inflammatory signaling genes with IL‐6‐induced recruitment of myeloid leukocytes to the pulmonary endothelium, inflammatory signaling, oxidative stress and vascular remodeling of the pulmonary endothelium. These findings were recapitulated in two experimental models of PH, one with known liver injury (MCT) but also in SuHx, a model not known to cause direct hepatic injury. There were concordant findings from human PAH PAECs with High‐MELD and the two experimental PH models with upregulation of cell survival genes and those related to hepatic growth factor signaling (*EGR1*). Finally, human liver tissue from PAH patients without liver disease or longstanding RV failure exhibited a pattern of fibrosis that was intermediate between controls and PoPH, mirroring the pattern seen in SuHx rats.

These findings reinforce prior work demonstrating subclinical liver injury predicts clinical outcomes in PAH patients who have participated in clinical trials (Scott et al. 2024). In this report, cholestatic liver injury identified with routine laboratory monitoring predicted worse outcomes, suggesting that bile acids and their metabolism play a role in lung‐liver communication in PAH. Circulating bile acids may influence pulmonary endothelial and lysosomal activity and cellular metabolism via the nuclear receptor coactivator 7 (NCOA7) (Harvey et al. 2025). In the MCT PH model, ketone metabolism is impaired in the liver and leads to activation of the NLRP3 inflammasome suggesting a link between aberrant hepatic metabolism and activation of a systemic inflammatory process (Blake et al. 2023), in line with our observations in both human and animal tissues. Taken together, these data support our hypothesis that the liver influences the pulmonary circulation as part of a feed‐forward loop before (and independent of) underlying liver disease and chronic hepatic congestion.

While specific mechanisms that link the hepatic and pulmonary vascular beds remain elusive, circulating immunologic and vasoactive factors that pass first through (or escape from) hepatic metabolism (Al‐Naamani and Roberts 2013) and vascular growth factors secreted by the liver are likely important. In children with congenital heart disease, creation of a cavopulmonary shunt causes pulmonary arteriovascular malformations (Duncan and Desai 2003; Freedom et al. 2004). Circulating BMPs, key signaling ligands in PAH (Guignabert et al. 2024) produced in the liver, are expressed at lower levels in patients with hepatopulmonary syndrome and portopulmonary hypertension as compared to liver disease controls (Owen et al. 2020; Rochon et al. 2020). Our network analysis identified a transcriptional signature in High‐MELD PAECs consistent with suppression of the BMP‐protective signaling arm and amplification of the opposing TGFβ/Activin axis, with *GREM1, COL3A1, LOX*, and *INHBA* upregulated in the same module (Cahill et al. 2012; Ghouleh et al. 2017; Ciuclan et al. 2013; Ryanto et al. 2021; Guignabert et al. 2023). Together, these findings suggest that subclinical liver dysfunction is associated with both reduced BMP bioavailability and increased Activin A production at the level of the pulmonary endothelium (Guignabert et al. 2024; Guignabert et al. 2023). While our observations require further experimental validation, this imbalance characterizes PAH pathobiology and is targeted by emerging therapies.

In human PAH PAECs with High‐MELD and PH rat lungs, we noted increased expression of *EGR1*. Hepatocyte growth factor (HGF) can induce the expression of *EGR1* to regulate cell cycle and proliferation (Ozen et al. 2012; Lee and Kim 2009) and in hepatocellular cancer, EGR1 is implicated in malignant cells' escape from anticancer drugs via stabilization of microtubules and autophagy (Peng et al. 2016; Peng et al. 2010). Increased HGF is associated with worse survival in PAH (Yang et al. 2016), however, HGF supplementation has been shown to be beneficial in experimental PH (Ono et al. 2004). Hepatokines may modulate endothelial dysfunction in PAH but require additional investigation. We also noted upregulation of gene expression related to cell proliferation (*ECM1*) (Han et al. 2001) and escape from cell death via Wnt signaling (*CTNNB1*) (Akiyama 2000; Clevers 2006), reinforcing well‐established paradigms of PAEC dysregulation in PAH (Chakraborty et al. 2023).

Macrophages are differentially polarized in PAH (Zawia et al. 2021; Luo and Qiu 2022), and depletion or inactivation of macrophages can prevent disease, including in portopulmonary hypertension (Krowka et al. 2012; Lazaro Salvador et al. 2021; Savale et al. 2020; Thenappan et al. 2011; Tian et al. 2013). PAECs are known to recruit leukocytes via increased expression of adhesion molecules (Le Hiress et al. 2015), consistent with our observation of upregulated gene programs related to leukocyte chemotaxis and myeloid leukocyte migration in High‐MELD PAECs (Radi et al. 2001; Vestweber 2007). Finally, cytokine signaling (e.g., IL‐6, TNF) was upregulated and were prominent hubs in High‐MELD PAECs concordant with established literature (Van Loo and Bertrand 2023; Kalliolias and Ivashkiv 2016).

This study has limitations. We cannot definitively exclude hepatic congestion as contributing to our findings, although we adjusted for right atrial pressure in our analysis to minimize confounding and where available, echocardiogram and invasive hemodynamics were used to exclude RV dysfunction in patients with liver biopsies. We acknowledge that the MELD‐Na was not developed for use in a population without liver disease nor does it comprehensively capture liver function; however, it has been used in to predict outcomes in left heart failure (Curcio et al. 2025). Our results were unchanged when we excluded connective tissue disease participants, who may be more prone to autoimmune liver disease and those taking warfarin, which may pharmacologically increase the INR (a component of MELD‐Na). We had similar results when we used alternative MELD scores (MELD‐Xi), and we have orthogonally validated our findings with the FIB‐4 index—an established noninvasive metric for liver stiffness (Shah et al. 2009). A sensitivity analysis evaluating individual components of MELD‐Na demonstrated that no single factor explained the relationship with PVR. The low MELD‐Na cluster was predominantly female (who had higher six‐minute walk distances), consistent with the known female advantage in PAH (Ventetuolo and Sherman‐Roe 2025). Furthermore, we have confirmed the hypotheses generated by our unsupervised analyses in two animal models of PH—one with known liver injury and one without—and human PAH, demonstrating hepatic inflammation and fibrosis is associated with pulmonary endothelial activation and activation of the pulmonary endothelium via expression of *VCAM‐1, TNF*, and *MIF*. Whether these observations in SuHx are due to Sugen5416 directly acting on the liver or pulmonary endothelial injury and lung‐liver crosstalk is unknown; time course experiments are planned for the future. However, VEGF inhibition in the liver has been shown to be antiproliferative and antifibrotic (Huang et al. 2013; Taniguchi et al. 2001), whereas we observed increased fibrosis and inflammation in SuHx. Any prior observations of liver injury in SuHx are in chronic RV failure models (Hamberger et al. 2022). While BMI may be a poor measure of body composition (Frankenfield et al. 2001), our conclusion that steatohepatitis is not a confounder in this study is supported by the lack of fatty liver infiltration on all available liver imaging. Human liver tissue samples were limited, but similar themes emerged across human and animal tissues. Failure to detect significant differences between the degree of fibrosis in PAH livers without liver disease (non‐PoPH) and control livers may have been due to sample size, and robust clinical data were not available for all participants. We identified approximately 20% collagen in control livers which is higher than previously described (Wang et al. 2023). This may be due to tissue being collected at the end of life; however, the relative increase in percent fibrosis in PAH subjects without liver disease remains notable. This hypothesis‐generating observation requires confirmation in a larger cohort.

## 5 Conclusion

In conclusion, we have presented novel observations of a lung‐liver axis characterized by inflammatory activation of the pulmonary endothelium associated with subclinical liver dysfunction. This relationship is characterized by hepatic inflammation and fibrosis, even in patients and animal models not known to have detectable liver disease. Taken together, this supports key interorgan modulation and a lung‐liver axis early in the PAH disease continuum.

## Author Contributions

N.S. and C.E.V. conceptualized the study and drafted the manuscript. N.S., C.J.M., M.P., A.S.‐R., A.T.J., T.C., J.R.K., W.O., and C.E.V. collected and propagated PAECs. J.L. and A.R. performed the cluster analysis of human PAEC bulk RNA sequencing. R.‐S.W. and N.S. performed the network analysis. J.H., S.B., and S.U. performed and analyzed rat liver bulk RNA sequencing. N.S. performed rat and analyzed rat and human histology. S.B., A.H., A.V., G.F., and S.U. performed human liver histology. Data analysis and interpretation was performed by N.S., R.‐S.W., O.D.L., and C.E.V. The manuscript was reviewed and approved by all authors.

## Funding

This work was completed with support from the National Institutes of Health R01‐HL141268 (C.E.V), R01‐HL174007 (C.E.V), U54‐GM115677‐S9 (PI: Rounds; C.E.V.); R01‐HL158841 (O.D.L, J.R.K), R01‐HL161038 (S.U.), P20‐GM103652 (E.O.H, C.E.V), T32‐HL134625 (N.S., E.O.H., C.E.V.), K08‐HL169982 (J.H.), the American Heart Association 24IPA1275127 (C.E.V), 24CDA1274310 (S.B.) and the American Thoracic Society (N.S.).

## Conflicts of Interest

N.S., J.L., A.R., S.B., A.H., J.H., C.J.M., M.P., A.S.‐R., A.T.J., T.C., G.F., J.R.K., W.O., R.‐S.W., Z.D., M.F., E.O.H., O.D.L., S.U., C.E.V. have declared that no conflicts of interest exist. C.E.V. has received personal fees from Merck & Co., Janssen Pharmaceuticals, Pulmovant and Regeneron, outside of the submitted work. Her institution has received fees for the conduct of clinical trials from Merck & Co., Pulmovant, Tenax, Gossamer and United Therapeutics. Patent application BM‐2024‐052; 15,024‐398PC0 pending to None.

## Supporting information


**Figure S1:** Flow diagram of patient screening, eligibility and exclusion for the study cohort. Among Group 1 PAH subjects, the diagnoses were independently confirmed and those with underlying liver disease (*n* = 1) were excluded. The final cohort consisted of 25 Group 1 PAH subjects without any clinically detectable liver disease. *PAH = pulmonary arterial hypertension*.
**Figure S2:** The FIB‐4 index provides orthogonal validation for the MELD‐Na score. (A) Scatterplot of MELD‐Na vs. FIB‐4 with spearman's correlation (*ρ* = 0.4, *p* = 0.045) in all participants demonstrates moderate correlation between the two non‐invasive scores. (B) There is a trend toward higher PVR in individuals with a FIB‐4 ≥ 2.67 (an established threshold for severe liver stiffness, Wilcoxon *p* = 0.17). *FIB‐4 = Fibrosis 4. MELD‐Na = model for end‐stage liver disease with sodium. PVR = pulmonary vascular resistance*.
**Table S1:** Network topology summary.
**Table S2:** Module summary.

## Data Availability

Raw and processed RNA sequencing data are available in the Gene Expression Omnibus under accession number GSE243193.
