# Supplementary material for: Exploring the Lung–Liver Axis in Pulmonary Arterial Hypertension
Source: Compr Physiol. 2026 May 15;16:e70171. doi: 10.1002/cph4.70171 (PMC13178415; doi:10.1002/cph4.70171)
Supplement: Supplementary file 1 — Figure S1: Flow diagram of patient screening, eligibility and exclusion for the study cohort. Among Group 1 PAH subjects, the diagnoses were independently confirmed and those with underlying liver disease (n = 1) were excluded. The final cohort consisted of 25 Group 1 PAH subjects without any clinically detectable liver disease. PAH = pulmonary arterial hypertension. Figure S2: The FIB‐4 index provides orthogonal validation for the MELD‐Na score. (A) Scatterplot of MELD‐Na vs. FIB‐4 with spearman's correlation (ρ = 0.4, p = 0.045) in all participants demonstrates moderate correlation between the two non‐invasive scores. (B) There is a trend toward higher PVR in individuals with a FIB‐4 ≥ 2.67 (an established threshold for severe liver stiffness, Wilcoxon p = 0.17). FIB‐4 = Fibrosis 4. MELD‐Na = model for end‐stage liver disease with sodium. PVR = pulmonary vascular resistance. Table S1: Network topology summary. Table S2: Module summary. [file CPH4-16-e70171-s001.docx]

**Exploring the Lung-Liver Axis in Pulmonary Arterial Hypertension**

Supplemental Materials

# Supplemental Methods

# Classification and Regression Tree (CART)

CART was selected for unsupervised stratification of patients by hepatic disease severity because it is a non-parametric method that does not assume linearity or normality, can handle a mix of continuous clinical variables, and produces interpretable decision rules with clinically meaningful thresholds. Right atrial pressure (RAP) is a strong determinant of pulmonary vascular resistance (PVR) in PAH patients with hepatic congestopathy and was therefore treated as a confounder rather than an independent variable of interest. To remove its contribution to PVR prior to CART analysis, a two-stage residualization approach was used. In Stage 1, a linear regression of PVR on RAP was fit, and the residuals (i.e., the variation in PVR unexplained by RAP) were extracted. In Stage 2, CART was applied to these residuals as the outcome variable. This approach ensures that any variable emerging as a splitting node in the tree is associated with PVR over and above the contribution of right heart filling pressure.

Independent variables entered into the CART model included MELD-Na score and other liver-focused clinical variables (e.g., AST, ALT, platelets, age, coded imaging data). The CART model was fit using the rpart package (v4.1) in R with method="anova", appropriate for a continuous outcome variable. The complexity parameter (cp) was left at its default value, as the anova method uses residual sum of squares reduction as the splitting criterion and cp primarily governs pruning in classification trees. The tree was allowed to grow without pre-specified depth constraints, with final partitions determined by the data.

Variable importance was extracted from the fitted CART model using the $variable.importance element, which reflects the total reduction in residual sum of squares attributable to each variable across all splits. The splitting threshold for MELD-Na was determined empirically by the tree algorithm (MELD-Na < 12 vs. ≥12) and was not pre-specified. Patients were classified as low or high MELD-Na group based on this data-driven threshold.

# Dimensionality Reduction: Principal Component Analysis via Singular Value Decomposition (PCA-SVD)

PCA was chosen to identify orthogonal axes of maximum variance in gene expression across patients, reducing the 62,000-dimensional feature space to a tractable number of components. PCA was performed using the prcomp() function in R, which employs Singular Value Decomposition (SVD) rather than traditional eigen decomposition of the variance-covariance matrix. All 25 principal components were retained and entered into downstream LDA modeling.

# Supervised Classification: Linear Discriminant Analysis (LDA)

LDA was used to determine whether gene expression-derived principal components could classify patients into MELD-Na groups (high vs. low) identified by CART. LDA was implemented using the lda() function from the MASS package in R, with MELD-Na group as the outcome and all 25 PCs as predictors. Prior probabilities were set proportional to group membership. To assess the contribution of individual components, univariate ANOVAs were performed for each PC with MELD-Na group as the independent variable. Components 9 and 21 emerged as the most discriminating. Classification accuracy was assessed using the model’s in-sample predictions, yielding an accuracy of 0.80.

# Differential Gene Expression: DESeq2

To identify a biologically interpretable gene set for downstream Random Forest modeling and network analysis, differential expression analysis between high and low MELD-Na groups was performed using DESeq2 (Bioconductor; Love et al., Genome Biology 2014). Raw counts were provided as input. Size factor normalization was performed internally by DESeq2 to account for differences in sequencing depth across samples. MELD-Na group (high vs. low) was specified as the design variable. 426 genes were excluded from FDR correction by DESeq2's independent filtering algorithm due to low mean normalized counts; no genes were flagged as count outliers by Cook's distance. Genes with nominal p < 0.05 were retained for downstream analysis (n = 2,458 genes) consistent with hypothesis-generating transcriptomic analyses in small PAH cohorts.

# Feature Selection and Classification: Random Forest

Random Forest was chosen as the primary outcomes model for gene expression classification because it is robust to high-dimensional data, handles correlated features without requiring variable selection *a priori*, and provides a built-in measure of variable importance. Unlike LDA, Random Forest does not assume multivariate normality or equal covariance across groups.

Random Forest models were implemented using the randomForest package in R with 500 trees (default) and importance=TRUE to enable variable importance estimation. Two models were fit: (1) a full model using all approximately 48,000 genes with no missing data, and (2) a reduced model using the 2,458 DESeq2-significant genes. Genes with any missing values were excluded from the full model.

Random Forest provides internal validation through Out-of-Bag (OOB) error estimation, which is a standard form of leave-one-out cross-validation inherent to the bootstrap sampling procedure used to grow each tree. Each tree is trained on approximately two-thirds of samples; the remaining one-third (out-of-bag samples) are used to estimate prediction error without requiring a held-out test set. The full model achieved an OOB accuracy of 0.56, while the DESeq2-filtered model achieved an OOB accuracy of 0.76, demonstrating that biologically informed feature selection substantially improved classification performance.

Gene importance was quantified using Mean Decrease in Accuracy (MDA), which reflects the reduction in classification accuracy when a given gene’s values are randomly permuted. Genes with MDA > 0 were retained as contributing predictors. Genes with zero or negative MDA were considered non-informative for MELD-Na group classification.

# Protein-Protein Interaction Network Analysis

To characterize the biological relationships among differentially expressed genes and identify key regulatory hubs, a protein-protein interaction (PPI) network analysis was performed. Protein-protein interaction network analysis was performed to identify functional relationships between differentially expressed genes and characterize the signaling pathways and molecular mechanisms coordinating transcriptomic differences between High- and Low-MELD clusters. Unlike pathway enrichment analysis, which tests predefined gene sets, PPI network analysis is data-driven and can reveal emergent biological structure, including hub genes that coordinate signaling across multiple pathways and functional modules that reflect shared biological programs. This approach was selected to address the question of which genes serve as central organizers of the transcriptomic response in High-MELD PAECs, and which biological processes are most coordinately regulated. The 2,458 DESeq2-significant genes were mapped to the STRING protein-protein interaction database (v12.0, Homo sapiens, species ID 9606) using the STRINGdb R package. Ensembl gene IDs were first converted to HGNC symbols using org.Hs.eg.db (Bioconductor) and AnnotationDbi. A combined confidence score threshold of ≥400 (medium confidence) was applied, reflecting interactions supported by multiple lines of evidence including experimental data, co-expression, text mining, and curated databases. A total of 1,409 genes (87.4%) mapped successfully to STRING identifiers; 361 genes (14.7%) were unmapped, typically representing poorly characterized genes or non-coding RNAs absent from the STRING database.

The resulting PPI network comprised 1,404 nodes and 7,406 edges. The largest connected component contained 1,259 genes (89.7% of mapped genes) and 7,393 interactions, with a network density of 0.0093. Mean node degree was 10.55 (median 5, maximum 132), consistent with scale-free network topology in which a small number of hub genes maintain disproportionately high connectivity. The node degree distribution followed a power law on a log-log scale, confirming scale-free architecture characteristic of biological PPI networks. Node-level metrics were calculated using the igraph R package and included: degree (number of direct interaction partners), normalized betweenness centrality (proportion of shortest paths between all node pairs traversing a given node), normalized closeness centrality (inverse mean shortest path length to all other nodes), and interaction strength (sum of edge weights). Hub genes were defined as nodes in the top 3rd percentile by betweenness centrality.

Network community structure was identified using the Louvain modularity optimization algorithm (set.seed = 42 for reproducibility), implemented in igraph. This algorithm partitions the network into densely interconnected modules by iteratively maximizing the modularity score Q, which measures the fraction of edges within modules relative to expectation under a null model. The analysis identified 16 modules (modularity Q = 0.554), well above the conventional threshold of Q > 0.3 for meaningful community structure. Module sizes ranged from 3 to 242 genes. Twelve modules containing ≥10 genes were carried forward for pathway enrichment analysis.

Gene Ontology Biological Process (GO:BP) enrichment analysis was performed for each module containing ≥10 genes using clusterProfiler (v4.12.6) with Benjamini-Hochberg FDR correction (significance threshold: FDR < 0.05). GO enrichment was additionally performed on Module 4 specifically, given its size (n = 242) and hub gene content. KEGG pathway enrichment was performed on all network genes. Ensembl gene IDs were mapped to Entrez IDs using org.Hs.eg.db (v3.19.1) and AnnotationDbi (v1.66.0). Network visualization was performed using ggraph (v2.2.2) with Fruchterman-Reingold force-directed layout; node size reflects betweenness centrality and node color reflects Louvain community membership.

# Software and Reproducibility

All analyses were performed in R (v4.4.1). Key packages: rpart (v4.1) for CART; FactoMineR and base prcomp() for PCA; MASS (v7.3-65) for LDA; DESeq2 (Bioconductor) for differential expression; randomForest for Random Forest; glmnet for penalized regression; irr for Cohen’s kappa; STRINGdb (v2.16.4) for PPI network construction; igraph (v2.2.2) for network topology; ggraph (v2.2.2) and ggplot2 (v4.0.2) for visualization; clusterProfiler (v4.12.6) for pathway enrichment; org.Hs.eg.db (v3.19.1) and AnnotationDbi (v1.66.0) for gene ID mapping; tidygraph (v1.3.1) for graph manipulation. Statistical analysis of clinical characteristics was performed in GraphPad Prism (v10.4.1). Analysis code is available upon request.

Excluded (n=1)

Portopulmonary Hypertension

Group 1 PAH patients without known liver disease (n=25)

Assessed for eligibility (n=26)

**Figure S1.** Flow diagram of patient screening, eligibility and exclusion for the study cohort. Among Group 1 PAH subjects, the diagnoses were independently confirmed and those with underlying liver disease (n=1) were excluded. The final cohort consisted of 25 Group 1 PAH subjects without any clinically detectable liver disease. *PAH=pulmonary arterial hypertension.*


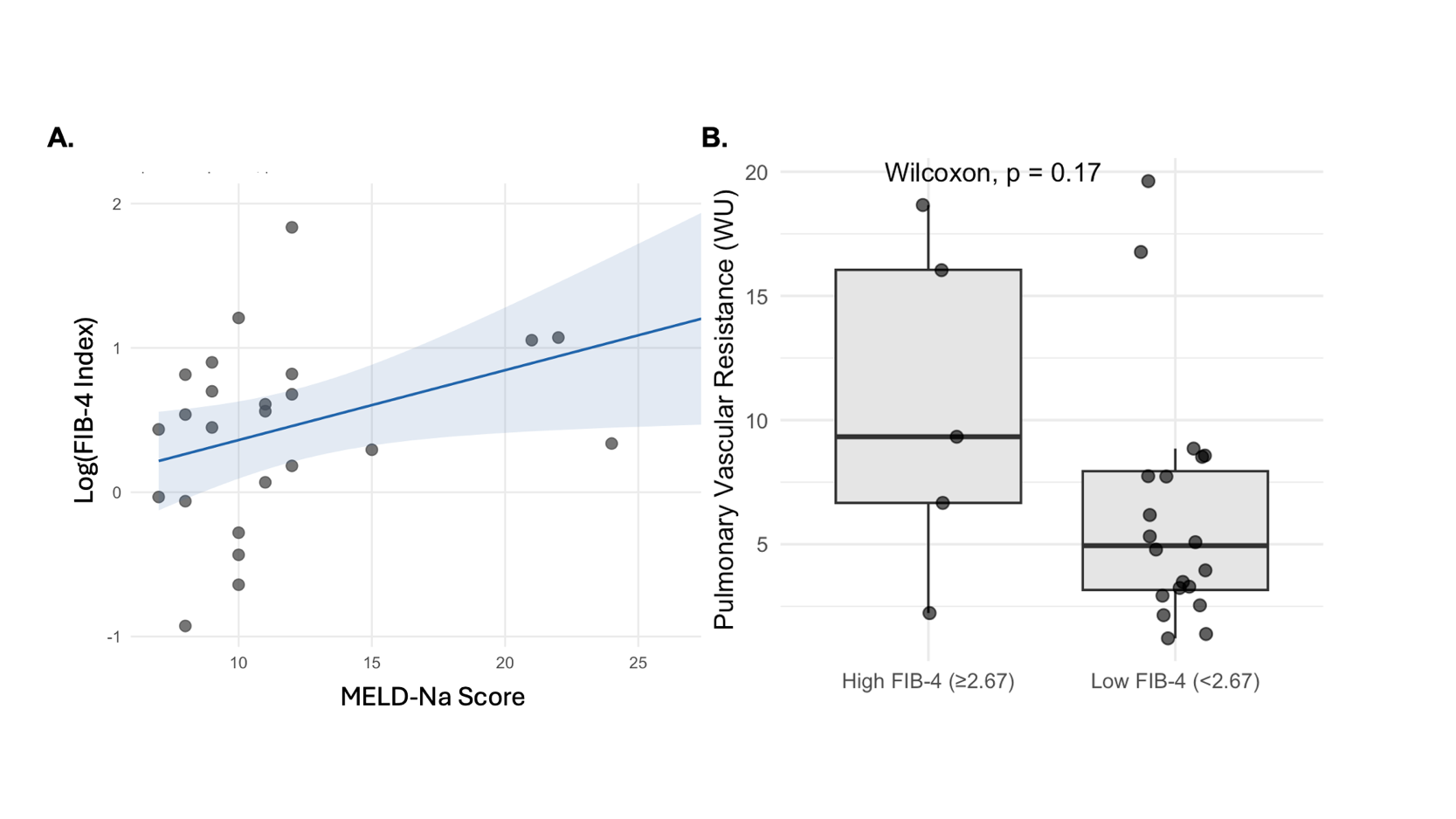


**Figure S2. The FIB-4 index provides orthogonal validation for the MELD-Na score. A.** Scatterplot of MELD-Na vs FIB-4 with spearman’s correlation (ρ=0.4, p=0.045) in all participants demonstrates moderate correlation between the two non-invasive scores. **B.** There is a trend toward higher PVR in individuals with a FIB-4 ≥2.67 (an established threshold for severe liver stiffness, Wilcoxon p=0.17). *FIB-4=Fibrosis 4. MELD-Na=model for end-stage liver disease with sodium. PVR=pulmonary vascular resistance.*

| **Table S1. Network Topology Summary** | |
| --- | --- |
| **Metric** | **Value** |
| Total nodes (genes) | 1404 |
| Total edges (interactions) | 7406 |
| Largest connected component - nodes | 1259 |
| Largest connected component - edges | 7393 |
| Network density | 0.0093 |
| Mean node degree | 11.74 |
| Median node degree | 6 |
| Maximum node degree (IL6) | 132 |
| Connected components | 133 |
| Louvain modules detected | 16 |
| Modularity score (Q) | 0.5543 |
| Nodes in visualized network (degree >= 5) | 771 |

| **Table S2. Module Summary** | | | | | | | |
| --- | --- | --- | --- | --- | --- | --- | --- |
| **Module** | **No. Genes** | **Genes Up** | **Genes Down** | **Mean LFC** | **Top Hub** | **Hub Betweenness** | **Hub LFC** |
| **4** | 242 | 197 | 45 | 1.10 | IL6 | 0.07 | 1.30 |
| **8** | 211 | 161 | 50 | 1.00 | CALML6 | 0.06 | 1.81 |
| **3** | 148 | 38 | 110 | -0.00 | TARDBP | 0.02 | -0.21 |
| **2** | 121 | 106 | 15 | 0.73 | CS | 0.03 | -0.31 |
| **13** | 114 | 61 | 53 | 0.35 | MED1 | 0.03 | -0.41 |
| **7** | 110 | 81 | 29 | 1.02 | LOX | 0.02 | 1.58 |
| **14** | 96 | 71 | 25 | 0.49 | HSP90AA1 | 0.05 | 0.61 |
| **11** | 70 | 39 | 31 | 0.28 | NEDD8 | 0.02 | 0.51 |
| **10** | 59 | 52 | 7 | 1.11 | COMT | 0.01 | 0.38 |
| **6** | 38 | 31 | 7 | 0.80 | PGM5 | 0.01 | 1.40 |
| **1** | 20 | 15 | 5 | 0.69 | CFAP221 | 0.01 | 2.07 |
| **9** | 11 | 8 | 3 | 1.12 | CD2AP | 0.01 | -0.53 |
| **5** | 6 | 5 | 1 | 0.74 | ACY1 | 0.00 | 0.71 |
| **12** | 6 | 6 | 0 | 1.57 | ANGPTL5 | 0.01 | 2.45 |
| **16** | 4 | 2 | 2 | -0.15 | HOXB5 | 0.00 | 1.61 |
| **15** | 3 | 2 | 1 | 0.41 | TIFA | 0.00 | -0.59 |
